# Supplementary material for: Analysis of PPARγ Signaling Activity in Psoriasis
Source: Int J Mol Sci. 2021 Aug 10;22(16):8603. doi: 10.3390/ijms22168603 (PMC8395241; doi:10.3390/ijms22168603)
Supplement: Supplementary file 1 [file ijms-22-08603-s001.zip › Supplemental materials_Analysis of PPARg signaling activity in psoriasis/Pathway models/Models images and html files/Model of PPARG related pathways in psoriasis (short version)/10908.html]

SRC


# Protein SRC

|  |  |
| --- | --- |
| URN | urn:agi-llid:6714 |
| Total Entities | 0 |
| Connectivity | 9981 |
| Name | SRC |
| Description | v-src avian sarcoma (Schmidt-Ruppin A-2) viral oncogene homolog |
| Notes | This gene is highly similar to the v-src gene of Rous sarcoma virus. This proto-oncogene may play a role in the regulation of embryonic development and cell growth. The protein encoded by this gene is a tyrosine-protein kinase whose activity can be inhibited by phosphorylation by c-SRC kinase. Mutations in this gene could be involved in the malignant progression of colon cancer. Two transcript variants encoding the same protein have been found for this gene. [provided by RefSeq, Jul 2008] |
| Primary Cell Localization | Cytoplasm |
| Class | Protein kinase |
| ObjectType | Protein |

---

|  |  |
| --- | --- |
| Pathway | Skin Aging |
|  | High Fat Diet Activates cAMP Related Exocytosis in Synapse and Endocrine Cell |
|  | Leptin and CCK8 Activate Nodose Ganglia Neuron |
|  | I-cell: CCK-8 Secretion and Regulation of Eating Behavior |
|  | Dopamine Receptors Signaling |
|  | Thyroid Hormones Common non-Genomic Effects in Hyperthyroidism |
|  | CXC Chemokine Receptor Signaling |
|  | CC Chemokine Receptor Signaling |
|  | Plasmin Effects in Inflammation |
|  | Platelet Activation via Adhesion Molecules |
|  | Proplatelet Maturation |
|  | Toll-like Receptor Independent Sterile Inflammation |
|  | Eat me Signal: Apoptotic Cell Induces Phagocytosis |
|  | Eicosanoids in Myeloid Cell Activation |
|  | Eosinophil Activation |
|  | Mast-Cell Activation without Degranulation |
|  | Mast-Cell Activation without Degranulation through KIT/F2R Signaling |
|  | Mast-Cells De Novo Synthesized Mediators via IgE Independent Signaling |
|  | CD16/CD14 Proinflammatory Monocyte Activation |
|  | anti-Inflammatory Function of Macrophage M2 Lineage |
|  | Neutrophil Chemotaxis |
|  | Vascular Smooth Muscle Cell/Pericyte Migration and Proliferation |
|  | Vascular Endothelial Cell Activation by Blood Coagulation Factors |
|  | Vascular Endothelial Cell Activation by Growth Factors |
|  | Vascular Endothelial Cell Permeability Activation |
|  | Potassium Flux Regulation |
|  | GFs/TNF -> Ion Channels |
|  | Nociception Expression Targets Signaling |
|  | Vascular Motility |
|  | Ca2+ Flux Regulation |
|  | ADRA2C/ADRB2 -> Vasoconstriction |
|  | ADRA2C/ADRB2 -> Synaptic Endocytosis |
|  | ADRB1 -> Prostaglandin Generation |
|  | DRD2 Expression Targets |
|  | DRD2 -> TRPC1 Transcription |
|  | DRD2/4 -> Membrane Transport |
|  | CHRM1 -> IL2 Production |
|  | OPRK -> Pain Perception |
|  | IL6 Expression Targets -> Nociception |
|  | GABA(B)R -> Postsynaptic Inhibition |
|  | NTRK1/2/3 -> Acetylcholine Production |
|  | EPHB -> NMDA Receptor Activation |
|  | Vitamine D Deficite and Dentin Formation |
|  | G0/G1 Phase Transition |
|  | Adherens Junction Assembly (Nectin) |
|  | Focal Junction Assembly |
|  | Adherens Junction Assembly (Cadherins) |
|  | Proteins Involved in Arterial Hypertension |
|  | Endothelin Signaling in Arterial Hypertension |
|  | Arterial Hypertension |
|  | Endothelial Cell Dysfunction in Arterial Hypertension |
|  | Aldosterone Synthesis Chronic Regulation |
|  | Smooth Muscle Cell Dysfunction in Arterial Hypertension |
|  | TRPM4/6/7/8 Signaling in Arterial Hypertension (Hypothesis) |
|  | Proteins Involved in Pulmonary Hypertension |
|  | Endothelial Cell Dysfunction in Pulmonary Hypertension |
|  | Myocardial Remodeling |
|  | Proteins Involved in Myocarditis |
|  | Vascular Reactivity in Raynaud Disease |
|  | Melanoma |
|  | Proteins Involved in Melanoma |
|  | Vitiligo |
|  | Apoptosis of Melanocyte in Vitiligo |
|  | Macrophage Function in Diabetes Mellitus Type 1 |
|  | Treg-Cell Activation in Diabetes Mellitus |
|  | Tubular Cell Dysfunction in Progressive Diabetic Nephropathy |
|  | Endothelial Cell Dysfunction in Progressive Diabetic Nephropathy |
|  | Mesangial Cell Dysfunction in Diabetic Nephropathy |
|  | Proliferative Diabetic Retinopathy |
|  | Hepatic Stellate Cells in non-Alcoholic Fatty Liver Disease |
|  | Hyperparathyroidism, Neonatal Severe |
|  | Bone Remodeling in Hyperthyroidism |
|  | KISS1 Production Impairment in Hypogonadotropic hypogonadism |
|  | Hypoparathyroidism, Primary |
|  | Proteins Involved in Obesity |
|  | Enterocyte Mediated Mucosal Damage in Celiac Disease |
|  | Proteins Involved in Colorectal Neoplasms |
|  | Metastatic Colorectal Cancer |
|  | GUCY2C Signaling in Colorectal Cancer |
|  | OipA Signaling in Helicobacter Pylori Infection |
|  | CagA Phosphorylation Independent Signaling |
|  | CagA Phosphorylation Dependent Signaling |
|  | B-Cell Acute Lymphoblastic Leukemia |
|  | B-Cell Chronic Lymphocytic Leukemia |
|  | Chronic Myeloid Leukemia |
|  | Proteins Involved in Chronic Myeloid Leukemia |
|  | Follicular Lymphoma |
|  | Hodgkin Lymphoma |
|  | Mucosa-Associated Lymphoid Tissue (MALT) Lymphoma |
|  | Osteoclast Activation in Multiple Myeloma |
|  | IL6/IGF1/VEGFA Signaling in Multiple Myeloma |
|  | Multiple Myeloma |
|  | Proteins Involved in Hepatocellular Carcinoma |
|  | Hepatocellular Carcinoma |
|  | Growth Factor Signaling in Hepatocellular Carcinoma |
|  | Proteins Involved in Pancreatic Cancer |
|  | Pancreatic Ductal Carcinoma |
|  | Growth Factor Signaling in Pancreatic Neoplasms |
|  | Pancreatic Neuroendocrine Tumors |
|  | Eosinophil Activation and Degranulation in Asthma |
|  | Airway Smooth Muscle Cell High Rate Proliferation |
|  | IgE Induces Airway Smooth Muscle Cell Proliferation |
|  | Airway Smooth Muscle Cell Contraction |
|  | Mast-Cell Related Chemokines Signaling in Asthma |
|  | Osteoclast Activation in Gout |
|  | Osteoclast Activation in Psoriatic Arthritis |
|  | Synovial Fibroblast Proliferation in Rheumatoid Arthritis |
|  | Osteoclast Activation in Rheumatoid Arthritis |
|  | Autocrine Cytokine/Chemokine Loops in Systemic Scleroderma |
|  | Skin Fibrosis |
|  | WHIM Syndrome |
|  | CCR5 Signaling in Macrophages |
|  | Macrophage Survival through CCR5 and CXCR4 Mediated Signaling |
|  | Estrogen Effects on Pregnancy Gingivitis |
|  | Hereditary Hemorrhagic Telangiectasia |
|  | Cowden Syndrome |
|  | Glutamate Toxiicity Effects on Striatum Medium Spiny Neurons in Hantington's Disease |
|  | Ca2+ Overload in Muscular Dystrophies |
|  | Muscular Dystrophies |
|  | Dystrophin Glycoprotein Complex Signaling in Muscular Dystrophies |
|  | Proteins Involved in Osteopetrosis |
|  | Proteins Involved in Osteoporosis |
|  | Osteoclast Activation in Postmenopause |
|  | Proteins Involved in Alzheimer's Disease |
|  | Proteins Involved in Epilepsy |
|  | BDNF Upregulation Triggered by Seizures |
|  | HMGB1 and IL1B in Neuronal Hyperexcitation |
|  | Glioblastoma, Primary |
|  | Medulloblastoma |
|  | Growth Factor Signaling in Neuroblastoma |
|  | Neuroblastoma |
|  | Proteins Involved in Neuroblastoma |
|  | Microglia and Motor Neuron Interaction Dysregulation |
|  | SPP1 Induced Survival of Myelin Specific T-Cells in Multiple Sclerosis |
|  | Proteins Involved in Breast Cancer Related to ESR1 Signaling Pathway |
|  | Proteins Involved in Breast Cancer Related to ERBB2/VEGFR/Akt Signaling Pathway |
|  | ERBB/VEGFR/Akt Signaling in Breast Cancer |
|  | IGF1R/AKT Signaling in Breast Cancer |
|  | ESR1/ERBB Positive Luminal Breast Cancer |
|  | Proteins with Altered Expression in Breast Cancer |
|  | Proteins Involved in Breast Cancer Related to IGF1R/Akt Signaling Pathway |
|  | Breast Cancer |
|  | ESR1 Signaling in Breast Cancer |
|  | Endometrial Cancer |
|  | Endometrioid Endometrial Cancer |
|  | Clear Cell Endometrial Cancer and Papillary Serous Endometrial Cancer |
|  | Angiogenesis in Endometriosis |
|  | Coagulation Factors and PAR1/2 Receptors (F2R/F2RL1) in Endometriosis |
|  | Proteins Involved in High-Grade Serous Ovarian Carcinoma |
|  | Endometrioid Ovarian Carcinoma |
|  | Proteins Involved in Endometrioid Ovarian Carcinoma |
|  | Clear Cell Ovarian Carcinoma |
|  | Proteins Involved in Mucinous Ovarian Carcinoma |
|  | High-Grade Serous Ovarian Carcinoma |
|  | Low-Grade Serous Ovarian Carcinoma |
|  | Proteins Involved in Low-Grade Serous Ovarian Carcinoma |
|  | Ovarian Cancer |
|  | Proteins Involved in Clear Cell Ovarian Carcinoma |
|  | Mucinous Ovarian Carcinoma |
|  | MIF Role in Cholesteatoma |
|  | Cholesteatoma |
|  | HPV E5 Regulates EDNRA and EGFR Signaling Pathway in Keratinocytes |
|  | Androgen Receptor Coregulator ARA55 (TGFB1I1) in Prostate Cancer |
|  | Prostate Cancer |
|  | TRPM8 Effects in Prostate Cancer (Hypothesis) |
|  | Proteins Involved in Prostate Cancer |
|  | Ca2+ Toxicity in Lens Cells |
|  | Proteins Involved in Cataract |
|  | VEGFA in Neovascular Glaucoma |
|  | Proteins Involved in Noise-Induced Hearing Loss |
|  | Alveolar Macrophages Dysfunction in COPD |
|  | VEGFA Low Expression Level in Chronic Bronchitis |
|  | VEGFA High Expression Level in Pulmonary Emphysema |
|  | T-Cell Cytotoxic Activation |
|  | Alveolar Epithelial Cell Dysfunction in COPD |
|  | Proteins Involved in Polycystic Kidney Disease |
|  | Proteins Involved in Glomerulonephritis |
|  | Plasmin Role in Glomerulonephritis |
|  | Endothelial Cell Dysfunction in Glomerulonephritis |
|  | Mesangial Cell Dysfunction in Glomerulonephritis |
|  | Interstitial Fibroblasts in Pyelonephritis |
|  | Sertoli Cells Dysfunction Causes Male Infertility |
|  | CD157/ITGB2 Signaling in Myeloid Cell |
|  | CD38 -> cADPR/Calcium Signaling |
|  | ANGPT1 -> CREB/CREBBP Expression Targets |
|  | ANGPT2 -> AP-1/CREBBP/MYC Expression Targets |
|  | AREG -> NFATC Expression Target |
|  | AREG -> CREB/CREBBP Expression Targets |
|  | AREG -> NCOR2 Expression Targets |
|  | AREG -> CTNN Expression Targets |
|  | AREG -> HIF1A Expression Targets |
|  | AREG -> STAT Expression Targets |
|  | AREG -> AP-1 Expression Targets |
|  | Noradrenaline/Gs Expression Targets |
|  | Epinephrine/Gs Expression Targets |
|  | Acetylcholine Expression Targets |
|  | Dopamine/Gi Expression Targets |
|  | Aryl Hydrocarbon Receptor Genomic and non-Genomic Signaling |
|  | BTC -> NFATC Expression Targets |
|  | BTC -> AP-1/ATF/CREB Expression Targets |
|  | BTC -> CTNN Expression Targets |
|  | BTC -> STAT Expression Targets |
|  | AGTR1 -> ARRB1/ARRB2 Signaling |
|  | INSR -> ARRB2/AKT/SRC Signaling |
|  | ARRB1/ARRB2 non-CanonicalSignaling and HedgehogFamily |
|  | CD72 -> NF-kB Expression Targets |
|  | CD8 -> NF-kB Expression Targets |
|  | CD80 -> NFATC Expression Targets |
|  | CD86 -> NFATC Expression Targets |
|  | EGF -> CTNN Expression Targets |
|  | EGF -> HIF1A Expression Targets |
|  | EGF -> AP-1/ATF Expression Targets |
|  | EGF -> CREB/CREBBP/ELK/SRF/MYC Expression Targets |
|  | EGF -> MEF/MYOD/NFATC Expression Targets |
|  | EGF -> TP53 Expression Targets |
|  | EGF -> NCOR2 Expression Target |
|  | EGF -> STAT Expression Targets |
|  | EGFR -> NCOR2 Signaling |
|  | EGFR/ERBB3 -> MEF/MYOD/NFATC/MYOG Signaling |
|  | EGFR -> CTNND Signaling |
|  | EGFR/ERBB2 -> TP53 Signaling |
|  | EGFR/ERBB2 -> CTNNB Signaling |
|  | EGFR/ERBB2 -> HIF1A Signaling |
|  | EGFR -> AP-1/ATF2 Signaling |
|  | EGFR -> AP-1/CREB/ELK/SRF/MYC Signaling |
|  | EGFR/ERBB -> STAT Signaling |
|  | EREG -> CREB Expression Target |
|  | EREG -> CTNNB/CTNND Expression Target |
|  | EREG -> STAT Expression Targets |
|  | EREG -> HIF1A Expression Target |
|  | EREG -> AP-1/ATF Expression Targets |
|  | Erythropoietin -> AP-1/MYC/CREB Expression Targets |
|  | F2 -> AP-1/CREB/ELK/SRF/SP1 Expression Targets |
|  | F2 -> STAT1/NF-kB Expression Targets |
|  | PLG -> AP-1/CREB/ELK/SRF/SP1 Expression Targets |
|  | PLG -> STAT1/NF-kB Expression Targets |
|  | FGF9 -> AP-1/CREB/MYC Expression Targets |
|  | FGF10 -> AP-1/CREB/CREBBP/MYC Expression Targets |
|  | FGF2 -> STAT Expression Targets |
|  | FGF1 -> AP-1/CREB/ELK/SRF/MYC Expression Targets |
|  | FGF1 -> NCOR2 Expression Target |
|  | FGF8 -> AP-1/CREB/MYC Expression Targets |
|  | FGF10 -> STAT Expression Targets |
|  | FGF18 -> AP-1/CREB Expression Targets |
|  | FGF23 -> NCOR2 Expression Targets |
|  | FGF1 -> STAT Expression Targets |
|  | FGF2 -> NCOR2 Expression Targets |
|  | FGF4 -> AP-1/MYC Expression Targets |
|  | FGF7 -> AP-1/CREB/CREBBP/MYC Expression Targets |
|  | FGF2 -> AP-1/CREB/CREBBP/ELK/SRF/MYC Expression Targets |
|  | FGFR1 -> STAT Signaling |
|  | FGFR -> AP-1/CREB/CREBBP/ELK/SRF/MYC Signaling |
|  | Glutamate/Gq Expression Targets |
|  | HBEGF -> STAT Expression Targets |
|  | HBEGF -> AP-1/ATF Expression Targets |
|  | HBEGF -> TP53 Expression Targets |
|  | HBEGF -> HIF1A Expression Targets |
|  | HBEGF -> MEF/MYOD Expression Target |
|  | HBEGF -> CREB/MYC Expression Targets |
|  | HGF -> STAT Expression Targets |
|  | HGF -> AP-1/CREB/ELK/SRF/MYC Expression Targets |
|  | HGFR -> STAT Signaling |
|  | HGFR -> AP-1/CREB/MYC Signaling |
|  | EGFR Transactivation in Cancer and Non-Cancer Cells |
|  | TAM Receptor Signaling Activation in Cancer |
|  | Receptors and Adaptor Proteins Activated in Cancer |
|  | Proteins with Altered Expression in Cancer-Associated Sustaining of Proliferative Signaling |
|  | G0/G1 Cell Cycle Phase Transition Activation in Cancer |
|  | STAT3 and NFkB Activate Inflammation-Induced Tumorigenesis |
|  | STAT3 Inhibits Maturation of Dendritic Cells and Facilitates Cancer Progression |
|  | VEGFA Dependent Angiogenesis in Cancer |
|  | VEGF Independent Angiogenesis in Cancer |
|  | Integrins in Cancer Cell Motility, Invasion and Survival |
|  | Proteins with Altered Expression in Cancer Metastases |
|  | Epithelial to Mesenchymal Transition in Cancer: Overview |
|  | Hyaluronic Acid, CD44 and HMMR in Cancer Cell Invasion and Survival |
|  | CDH2 Activation Promotes Cancer Cell Migration and Survival |
|  | CFL1 in Cancer Cell Motility |
|  | Invadopodia Formation in Cancer Cells |
|  | Myeloid Derived Suppressor Cells in Cancer Immune Escape |
|  | IGF1 -> MEF/MYOD/MYOG Expression Targets |
|  | IGF2/MEF/MYOD Expression Targets |
|  | IGF2 -> HIF1A/MYC Expression Targets |
|  | IGF1 -> ELK/SRF/HIF1A/MYC/SREBF Expression Targets |
|  | Insulin -> MEF/MYOD Expression Targets |
|  | Insulin -> ELK/SRF/HIF1A/MYC/SREBF Expression Targets |
|  | PECAM -> STAT Signaling |
|  | IGF1R -> ELK/SRF/HIF1A/MYC/SREBF Signaling |
|  | IGF1R -> MEF/MYOD/MYOG Signaling |
|  | FibronectinR -> AP-1/ELK/SRF/SREBF Signaling |
|  | MacrophageR -> CEBPB -> NF-kB Signaling |
|  | CTGF -> AP-1/CREB/MYC Expression Targets |
|  | CTGF -> NCOR2 Expression Target |
|  | ICAM1 Expression Targets |
|  | Fibrinogen Expression Targets |
|  | IL6ST -> STAT5B Signaling |
|  | IL6 Expression Targets |
|  | IL16-> ATF/CREB/CREBBP Expression Target |
|  | Morphine Expression Targets |
|  | Proteins/Ions Transporters -> Expression Targets in Lymphoid System and Blood |
|  | NRG1 -> MEF/MYOD Expression Targets |
|  | NRG1 -> CREB/CREBBP/ELK/SRF/MYC Expression Targets |
|  | NRG1 -> TP53 Expression Target |
|  | NRG1/Catenin Expression Targets |
|  | NRG1 -> STAT Expression Targets |
|  | NRG1 -> AP-1/ATF Expression Targets |
|  | NRG1 -> HIF1A Expression Target |
|  | NGF -> SMAD3/NF-kB Expression Targets |
|  | NGF -> AP-1/TP53/MYC Expression Targets |
|  | NGF -> FOXO/MYCN/ELK-SRF Expression Targets |
|  | BDNF Expression Targets |
|  | NGF -> CREB/CEBPB/MEF2A Expression Targets |
|  | Adenosine Expression Targets |
|  | PDGFC -> STAT Expression Target |
|  | PDGFB -> AP-1/CREB/MYC Expression Target |
|  | PDGFC -> CREB Expression Target |
|  | PDGFD -> AP-1 Expression Targets |
|  | PDGFD -> STAT Expression Targets |
|  | PDGF -> AP-1/CREB/CREBBP/MYC Expression Targets |
|  | PDGF -> STAT Expression Targets |
|  | PDGFB -> STAT Expression Target |
|  | KIT -> MITF Signaling |
|  | PDGFR -> STAT Signaling |
|  | CSF2 -> STAT Signaling |
|  | PDGFR -> AP-1/MYC Signaling |
|  | AVP/Gs -> CREB/ELK/SRF/AP-1/EGR Expression Targets |
|  | POMC Expression Targets |
|  | AVP/Gs -> MEF/MYOD/NFATC/MYOG Expression Targets |
|  | AVP/Gq -> STAT Expression Targets |
|  | IFNA1/Gq Expression Targets |
|  | AVP/Gs -> STAT Expression Targets |
|  | AVP/Gq -> MEF/MYOD/NFATC/MYOG Expression Targets |
|  | AVP/Gq -> CREB/ELK/SRF/AP-1/EGR Expression Targets |
|  | PDCD1 -> ATF/CREB/CREBBP Expression Targets |
|  | AngiopoietinR -> AP-1 Signaling |
|  | EphrinB -> Cytoskeleton Signaling |
|  | GDNF -> HSF1 Signaling |
|  | EphrinB -> MAPK/JUN/FOS Signaling |
|  | NTRK -> FOXO/MYCN Signaling |
|  | NTRK -> AP-1/CREB/ELK/SRF/MYC/SMAD3/TP53 Signaling |
|  | CSF1 -> STAT Expression Targets |
|  | GAS6 -> AP-1/CREB Expression Targets |
|  | FIGF -> AP-1 Expression Target |
|  | FIGF -> NCOR2 Expression Target |
|  | KITLG -> AP-1/CREB/CREBBP/MYC Expression Targets |
|  | GDNF -> HSF1 Expression Targets |
|  | CSF1 -> AP-1/CREB/CREBBP/MYC Expression Targets |
|  | PGF -> AP-1/CREB/CREBBP/MYC Expression Targets |
|  | FLT3LG -> AP-1/CREB/CREBBP Expression Targets |
|  | Ras-GRF Regulation Signaling |
|  | Androgen Receptor non-Genomic Signaling |
|  | FOXO1 Signaling |
|  | MERTK Signaling |
|  | Ras-GAP Regulation Signaling |
|  | PI3K/RAC1 Signaling |
|  | VEGF Signaling |
|  | Estrogens/ESR1 non-Genomic Signaling |
|  | EGFR Signaling |
|  | ERK5/MAPK7 Signaling |
|  | ERK/MAPK Canonical Signaling |
|  | TCR -> CREB/CREBBP/ATF Expression Targets |
|  | TCR -> NFAT Expression Targets |
|  | TCR -> AP-1 Expression Targets |
|  | TGFA -> STAT Expression Targets |
|  | TGFA -> CTNNB/CTNND Expression Targets |
|  | TGFA -> TP53 Expression Targets |
|  | TGFA -> HIF1A Expression Targets |
|  | TGFA -> MEF/MYOD/NFATC Expression Targets |
|  | TGFA/AP-1/ATF Expression Targets |
|  | TGFA -> CREB/CREBBP/ELK-SRF/MYC Expression Targets |
|  | CD40LG -> NF-kB/ELK/SRF -> CREB/NFATC Expression Targets |
|  | TNFSF13B Expression Targets |
|  | TNFRSF5/13B -> NFATC1 Signaling |
|  | Thrombopoietin -> AP-1/CREB/CREBBP/MYC Expression Targets |
|  | ROS in Angiotensin Mediated Cardiovascular Remodeling and Hypertrophy |
|  | VEGFA -> ATF/CREB/ELK-SRF Expression Targets |
|  | VEGFC -> CTNNB Expression Target |
|  | VEGFA -> NFATC Expression Targets |
|  | VEGFA -> STAT Expression Targets |
|  | VEGFC -> ATF Expression Target |
|  | VEGFA -> AP-1/CREBBP/MYC Expression Targets |
|  | VEGFA -> CTNNB/CTNND Expression Targets |
|  | VEGFA -> NCOR2 Expression Target |
|  | VEGFR -> CTNND Signaling |
|  | VEGFR -> CTNNB Signaling |
|  | VEGFR -> NFATC Signaling |
|  | VEGFR -> STAT Signaling |
|  | VEGFR -> AP-1/CREB/MYC Signaling |
|  | VEGFR -> ATF/CREB/ELK-SRF Signaling |
|  | WNT5A Expression Targets |
|  | Model of PPARG signaling in psoriasis |
|  | PPARG negative regulators and targets |
|  | Model of PPARG related pathways in psoriasis (short version) |
|  | 1\_Osteoclast-mediated bone resorption in postmenopausal women |
|  | 2\_Osteoblast-mediated bone demineralization |
|  | 3\_The role of interstitial fibroblasts in pyelonephritis |
|  | 3\_Mesangial cell dysfunction in glomerulonephritis |
|  | 1\_Local estrogen production stimulates ectopic endometrium proliferation |
|  | 3\_Enhanced angiogenesis in endometriosis |
|  | 1\_Disruption of luteinizing hormone and follicle-stimulating hormone secretion in PCOS |
|  | 1\_Endothelial cells stimulate vasoconstriction and thrombosis in pulmonary hypertension |
|  | 1\_Cigarette smoke causes dysfunction and production of inflammatory mediators in alveolar epithelial cells |
|  | 3\_Role of alveolar macrophages in pulmonary tissue destruction in COPD |
|  | 2\_1\_Eosinophilia and neutrophilia in asthma: eosinophil migration and activation |
|  | 4\_1\_Airway smooth muscle cells contraction |
|  | 4\_2\_Airway smooth muscle cells proliferation and airway tissue remodeling |
|  | 1\_CD4+ T cell and epithelial cell response in celiac disease |
|  | 2\_1\_General pathways of thyroid hormone signaling |
|  | drug-target moltransport |
|  | Model of PPARG signaling in psoriais (tested) |
|  | mapk1 protein modificators |

---

|  |  |
| --- | --- |
| Group | Proteins Involved in B-Cell Acute Lymphoblastic Leukemia |
|  | Proteins Involved in Cowden Syndrome |

---

|  |  |
| --- | --- |
| Source | curated |
|  | ResNet |
|  | GO component |

---

|  |  |
| --- | --- |
| MedScan ID | 6714 |

---

|  |  |
| --- | --- |
| LocusLink ID | 6714 |
|  | 20779 |
|  | 83805 |
|  | 99351 |
|  | 280929 |
|  | 535742 |
|  | 325084 |

---

|  |  |
| --- | --- |
| Alias | tyrosine kinase pp60c-src |
|  | SRC proto-oncogene, nonreceptor tyrosine kinase |
|  | proto-oncogene c-Src |
|  | tyrosine protein kinase c-src |
|  | SRC proto-oncogene, non receptor tyrosine kinase |
|  | SRC |
|  | Rous sarcoma oncogene |
|  | SRC-1 kinase |
|  | Schmidt-Ruppin A-2 viral oncogene homolog |
|  | Src protein kinase |
|  | neuronal proto-oncogene tyrosine-protein kinase Src |
|  | v-src sarcoma (Schmidt-Ruppin A-2) viral oncogene homolog |
|  | RP5-823N20.1 |
|  | Protooncogene Src, Rous sarcoma |
|  | p60-Src |
|  | pp60c-src |
|  | protooncogene src |
|  | OTTHUMP00000174478 |
|  | tyrosine kinase SRC-1 |
|  | c-Src |
|  | RP23-169M4.1 |
|  | Src tyrosine kinase |
|  | v-src sarcoma (Schmidt-Ruppin A-2) viral oncogene homolog (avian) |
|  | OTTMUSP00000019027 |
|  | p60c-src |
|  | proto-oncogene Src |
|  | OTTHUMP00000174476 |
|  | SRC I |
|  | OTTMUSP00000017316 |
|  | OTTHUMP00000174477 |
|  | v-Src |
|  | Src PTK |
|  | OTTMUSP00000019029 |
|  | c-Src tyrosine kinase |
|  | THC6 gene |
|  | v-src avian sarcoma (Schmidt-Ruppin A-2) viral oncogene homolog |
|  | Proto-oncogene tyrosine-protein kinase Src |
|  | SRC1 |
|  | OTTMUSP00000019028 |
|  | ASV |
|  | THC6 |
|  | tyrosine-protein kinase SRC-1 |
|  | AW259666 |
|  | tyrosine protein kinase pp60-c-src |
|  | SRC proto-oncogene, non-receptor tyrosine kinase |
|  | Src protein tyrosine kinase |
|  | expressed sequence AW259666 |
|  | Schmidt-Ruppin A-2) viral oncogene homolog |
|  | SRC SH2-domain |
|  | SRC SH3-domain |
|  | tyrosine protein kinase SRC-1 |
|  | chromosome |
|  | fc54g04 |
|  | protein-tyrosine kinase |
|  | scaffold |
|  | wu:fc54g04 |
|  | Src N1 |

---

|  |  |
| --- | --- |
| EC Number | 2.7.10.2 |
|  | 2.7.10.1 |
|  | 2.7.1.112 |

---

|  |  |
| --- | --- |
| GO ID | 0005524 |
|  | 0070700 |
|  | 0042169 |
|  | 0005070 |
|  | 0045296 |
|  | 0071253 |
|  | 0019899 |
|  | 0046875 |
|  | 0030331 |
|  | 0070851 |
|  | 0020037 |
|  | 0005158 |
|  | 0005178 |
|  | 0044325 |
|  | 0016301 |
|  | 0019900 |
|  | 0004715 |
|  | 0051219 |
|  | 0008022 |
|  | 0005080 |
|  | 0004672 |
|  | 0004713 |
|  | 0097110 |
|  | 0005102 |
|  | 0031625 |
|  | 0038128 |
|  | 0038096 |
|  | 0007186 |
|  | 0031295 |
|  | 0032148 |
|  | 0034332 |
|  | 0086098 |
|  | 0007411 |
|  | 0045453 |
|  | 0060444 |
|  | 0007049 |
|  | 0030154 |
|  | 0008283 |
|  | 0098609 |
|  | 0071398 |
|  | 0071498 |
|  | 0070301 |
|  | 0071456 |
|  | 0032869 |
|  | 0071222 |
|  | 0071375 |
|  | 0036120 |
|  | 0071393 |
|  | 0035635 |
|  | 0048013 |
|  | 0007173 |
|  | 0030900 |
|  | 0007229 |
|  | 0035556 |
|  | 0050900 |
|  | 0016236 |
|  | 2000811 |
|  | 0043066 |
|  | 0043154 |
|  | 2001237 |
|  | 0051895 |
|  | 2001243 |
|  | 0051902 |
|  | 0032463 |
|  | 0051974 |
|  | 0032211 |
|  | 0045892 |
|  | 0048011 |
|  | 0042476 |
|  | 0048477 |
|  | 0036035 |
|  | 0018105 |
|  | 0038083 |
|  | 0018108 |
|  | 0030168 |
|  | 2000573 |
|  | 0070374 |
|  | 0043406 |
|  | 0043065 |
|  | 0090263 |
|  | 0045737 |
|  | 0050715 |
|  | 0035306 |
|  | 0010634 |
|  | 0010907 |
|  | 0046628 |
|  | 0033625 |
|  | 2000394 |
|  | 1903997 |
|  | 2000386 |
|  | 0050731 |
|  | 0043552 |
|  | 0014068 |
|  | 2000588 |
|  | 0071803 |
|  | 0031954 |
|  | 0051897 |
|  | 1900182 |
|  | 0010954 |
|  | 0071902 |
|  | 0051057 |
|  | 0014911 |
|  | 0045893 |
|  | 0001545 |
|  | 0050847 |
|  | 0046777 |
|  | 0031648 |
|  | 0045124 |
|  | 2001286 |
|  | 0060491 |
|  | 0022407 |
|  | 2000641 |
|  | 0010632 |
|  | 0033146 |
|  | 0098962 |
|  | 0043393 |
|  | 0043114 |
|  | 0010447 |
|  | 0051602 |
|  | 0070555 |
|  | 0009612 |
|  | 0051385 |
|  | 0031667 |
|  | 0009615 |
|  | 0007172 |
|  | 0007165 |
|  | 0002223 |
|  | 0043149 |
|  | 0034446 |
|  | 0045056 |
|  | 0007179 |
|  | 0007169 |
|  | 0060065 |
|  | 0048010 |
|  | 0016032 |
|  | 0005884 |
|  | 0005901 |
|  | 0005737 |
|  | 0005829 |
|  | 0070062 |
|  | 0031234 |
|  | 0098978 |
|  | 0005770 |
|  | 0005764 |
|  | 0005743 |
|  | 0005739 |
|  | 0043005 |
|  | 0005634 |
|  | 0048471 |
|  | 0005886 |
|  | 0002102 |
|  | 0014069 |
|  | 0099091 |
|  | 0032587 |
|  | 0050839 |
|  | 0000166 |
|  | 0019904 |
|  | 0019901 |
|  | 0044877 |
|  | 0016740 |
|  | 0007155 |
|  | 0016477 |
|  | 0034614 |
|  | 0071560 |
|  | 0002376 |
|  | 0016310 |
|  | 0045785 |
|  | 0010628 |
|  | 1902533 |
|  | 0051222 |
|  | 0006468 |
|  | 0005856 |
|  | 0016020 |
|  | 1905587 |
|  | 0010641 |
|  | 0042493 |
|  | 0070542 |
|  | 0042542 |
|  | 0051427 |
|  | 0007417 |
|  | 0045087 |
|  | 0030520 |
|  | 0048008 |
|  | 0051726 |
|  | 0042127 |
|  | 0071801 |
|  | 0016337 |
|  | 0032403 |
|  | 0007265 |
|  | 0007596 |
|  | 0008543 |
|  | 0061024 |
|  | 0016772 |
|  | 0005622 |
|  | 0019048 |
|  | 0016044 |
|  | 0007243 |
|  | 0010740 |
|  | 0001101 |
|  | 0006950 |
|  | 0034329 |
|  | 0071445 |
|  | 0044419 |
|  | 0019717 |
|  | 0008152 |
|  | 0005515 |
|  | 0016021 |
|  | 0004674 |
|  | 0000074 |
|  | 0004872 |
|  | 0007242 |
|  | 0050875 |
|  | 0046854 |
|  | 0046934 |

---

|  |  |
| --- | --- |
| KEGG ID | hsa:6714 |
|  | mmu:20779 |
|  | rno:83805 |

---

|  |  |
| --- | --- |
| MedLine Reference | 11249956 |
|  | 11834728 |
|  | 11913791 |
|  | 12007793 |
|  | 12011049 |
|  | 12081562 |
|  | 12488346 |
|  | 12692262 |
|  | 12695509 |
|  | 12767047 |
|  | 12819032 |
|  | 12826681 |
|  | 14523024 |
|  | 14630916 |
|  | 14699011 |
|  | 14751567 |
|  | 14985360 |
|  | 15007081 |
|  | 15322113 |
|  | 15486073 |
|  | 15546918 |
|  | 15591141 |
|  | 15644319 |
|  | 15695822 |
|  | 15699470 |
|  | 15731459 |
|  | 15735685 |
|  | 15833739 |
|  | 15854740 |
|  | 15856020 |
|  | 15869794 |
|  | 15878970 |
|  | 15890643 |
|  | 16123104 |
|  | 16236484 |
|  | 16478473 |
|  | 16529858 |
|  | 16554304 |
|  | 16597920 |
|  | 16600505 |
|  | 16767106 |
|  | 16893669 |
|  | 16943426 |
|  | 17088251 |
|  | 10228160 |
|  | 10334875 |
|  | 10349636 |
|  | 10359184 |
|  | 10369734 |
|  | 10441734 |
|  | 10544143 |
|  | 10597276 |
|  | 10635317 |
|  | 11042159 |
|  | 11062345 |
|  | 11076861 |
|  | 11114734 |
|  | 11157779 |
|  | 11217851 |
|  | 11245687 |
|  | 11274221 |
|  | 11312300 |
|  | 11344051 |
|  | 11389024 |
|  | 11826110 |
|  | 11847100 |
|  | 11871859 |
|  | 11934902 |
|  | 11940607 |
|  | 11994463 |
|  | 11994738 |
|  | 12089510 |
|  | 12393559 |
|  | 12419774 |
|  | 12466851 |
|  | 12477932 |
|  | 12526739 |
|  | 12526740 |
|  | 12595302 |
|  | 12612063 |
|  | 12663375 |
|  | 12808090 |
|  | 12829028 |
|  | 12842760 |
|  | 12893822 |
|  | 12912979 |
|  | 12959979 |
|  | 1302014 |
|  | 1358284 |
|  | 1383278 |
|  | 1421760 |
|  | 14500722 |
|  | 14559149 |
|  | 14593208 |
|  | 14612570 |
|  | 14636584 |
|  | 14739300 |
|  | 14741371 |
|  | 15062102 |
|  | 1508191 |
|  | 15169891 |
|  | 15226823 |
|  | 15254245 |
|  | 15254900 |
|  | 15273716 |
|  | 15314167 |
|  | 15336554 |
|  | 15504909 |
|  | 15541355 |
|  | 15564375 |
|  | 15572128 |
|  | 15591224 |
|  | 15716419 |
|  | 15749833 |
|  | 15753384 |
|  | 15761030 |
|  | 15782199 |
|  | 15814670 |
|  | 15872089 |
|  | 15921498 |
|  | 1594760 |
|  | 16000375 |
|  | 16007215 |
|  | 16020549 |
|  | 16107610 |
|  | 1612587 |
|  | 16132110 |
|  | 16141072 |
|  | 16141073 |
|  | 16162939 |
|  | 16262245 |
|  | 16267818 |
|  | 16298995 |
|  | 16377083 |
|  | 16438943 |
|  | 16477001 |
|  | 16511561 |
|  | 16537894 |
|  | 16543952 |
|  | 16571868 |
|  | 16585062 |
|  | 16601135 |
|  | 16687575 |
|  | 16707485 |
|  | 16723512 |
|  | 16728403 |
|  | 16825188 |
|  | 16859680 |
|  | 16882656 |
|  | 16884713 |
|  | 17030621 |
|  | 1710116 |
|  | 17105771 |
|  | 1970329 |
|  | 1997203 |
|  | 205879 |
|  | 2404338 |
|  | 2440106 |
|  | 2570033 |
|  | 2575583 |
|  | 2759422 |
|  | 3291115 |
|  | 6246487 |
|  | 6255214 |
|  | 6320193 |
|  | 6328281 |
|  | 7507074 |
|  | 7512695 |
|  | 7530829 |
|  | 7539296 |
|  | 7542761 |
|  | 7617039 |
|  | 7685105 |
|  | 7689855 |
|  | 7846766 |
|  | 7909754 |
|  | 7911089 |
|  | 7958873 |
|  | 7959737 |
|  | 7961761 |
|  | 8001130 |
|  | 8034307 |
|  | 8095010 |
|  | 8145850 |
|  | 8270638 |
|  | 8634424 |
|  | 8661722 |
|  | 8672127 |
|  | 8690046 |
|  | 8756343 |
|  | 8812502 |
|  | 8849724 |
|  | 8858346 |
|  | 8983085 |
|  | 9006082 |
|  | 9062339 |
|  | 9160879 |
|  | 9195991 |
|  | 9257949 |
|  | 9315092 |
|  | 9325263 |
|  | 9344858 |
|  | 9353253 |
|  | 9373037 |
|  | 9374500 |
|  | 9488729 |
|  | 9658338 |
|  | 9687503 |
|  | 10070972 |
|  | 10075741 |
|  | 10092678 |
|  | 10187839 |
|  | 10195142 |
|  | 10205168 |
|  | 10206955 |
|  | 10339567 |
|  | 10358079 |
|  | 10362724 |
|  | 10391903 |
|  | 10395945 |
|  | 10455176 |
|  | 10455194 |
|  | 10458595 |
|  | 10462375 |
|  | 10467411 |
|  | 10482988 |
|  | 10527887 |
|  | 10544291 |
|  | 10635328 |
|  | 10657632 |
|  | 10681522 |
|  | 10688651 |
|  | 10698938 |
|  | 10734107 |
|  | 10777539 |
|  | 10777553 |
|  | 10787408 |
|  | 10838070 |
|  | 10846184 |
|  | 10858437 |
|  | 10871840 |
|  | 10884433 |
|  | 10899172 |
|  | 10918587 |
|  | 10931187 |
|  | 10945997 |
|  | 10959835 |
|  | 10971656 |
|  | 10973497 |
|  | 10978326 |
|  | 11007774 |
|  | 11013230 |
|  | 11024032 |
|  | 11039464 |
|  | 11071635 |
|  | 11106497 |
|  | 11113628 |
|  | 11127814 |
|  | 11152665 |
|  | 11279024 |
|  | 11279199 |
|  | 11279201 |
|  | 11294897 |
|  | 11309200 |
|  | 11313464 |
|  | 11350938 |
|  | 11353545 |
|  | 11373296 |
|  | 11375989 |
|  | 11379820 |
|  | 11382764 |
|  | 11445557 |
|  | 11481331 |
|  | 11483589 |
|  | 11483655 |
|  | 11514593 |
|  | 11524430 |
|  | 11593413 |
|  | 11602604 |
|  | 11708798 |
|  | 11713277 |
|  | 11723131 |
|  | 11724572 |
|  | 11739737 |
|  | 11741599 |
|  | 11741929 |
|  | 11751924 |
|  | 11781820 |
|  | 11834516 |
|  | 11854294 |
|  | 11872746 |
|  | 11875070 |
|  | 11877451 |
|  | 11884411 |
|  | 11923305 |
|  | 11927607 |
|  | 11934669 |
|  | 11940572 |
|  | 11964172 |
|  | 11971983 |
|  | 11983694 |
|  | 11994743 |
|  | 12011079 |
|  | 12014986 |
|  | 12048194 |
|  | 12052863 |
|  | 12060669 |
|  | 12063167 |
|  | 12091389 |
|  | 12095943 |
|  | 12145209 |
|  | 12153558 |
|  | 12162444 |
|  | 12210743 |
|  | 12215529 |
|  | 12216109 |
|  | 12219031 |
|  | 12235291 |
|  | 12244095 |
|  | 12387813 |
|  | 12393736 |
|  | 12402152 |
|  | 12415108 |
|  | 12416028 |
|  | 12420216 |
|  | 12429742 |
|  | 12429743 |
|  | 12429837 |
|  | 12454018 |
|  | 12456372 |
|  | 12462387 |
|  | 12468645 |
|  | 12473651 |
|  | 12493773 |
|  | 12522270 |
|  | 12538589 |
|  | 12540842 |
|  | 12540962 |
|  | 12554790 |
|  | 12576423 |
|  | 12588871 |
|  | 12604776 |
|  | 12615930 |
|  | 12618767 |
|  | 12621061 |
|  | 12624098 |
|  | 12626508 |
|  | 12631284 |
|  | 12637538 |
|  | 12649275 |
|  | 12707358 |
|  | 12730241 |
|  | 12753909 |
|  | 12771149 |
|  | 12789267 |
|  | 12795607 |
|  | 12808100 |
|  | 12810717 |
|  | 12829810 |
|  | 12846735 |
|  | 12878163 |
|  | 12907754 |
|  | 12932824 |
|  | 12933816 |
|  | 12939401 |
|  | 12947321 |
|  | 12972425 |
|  | 12975382 |
|  | 1310678 |
|  | 1379745 |
|  | 1383690 |
|  | 1384654 |
|  | 14504278 |
|  | 14517306 |
|  | 14551213 |
|  | 14562045 |
|  | 14617636 |
|  | 14627343 |
|  | 14661060 |
|  | 14670955 |
|  | 14676843 |
|  | 14687660 |
|  | 14688263 |
|  | 14702039 |
|  | 14706618 |
|  | 14707140 |
|  | 14739293 |
|  | 14761972 |
|  | 14766744 |
|  | 14963038 |
|  | 15001550 |
|  | 15033452 |
|  | 15060621 |
|  | 15062576 |
|  | 15069201 |
|  | 15075377 |
|  | 15094065 |
|  | 15117969 |
|  | 15122338 |
|  | 15143158 |
|  | 15175272 |
|  | 15184363 |
|  | 15229287 |
|  | 15271991 |
|  | 15272013 |
|  | 15276210 |
|  | 15286700 |
|  | 15297464 |
|  | 15304482 |
|  | 15337529 |
|  | 15342783 |
|  | 15363460 |
|  | 15456896 |
|  | 1545818 |
|  | 15467744 |
|  | 15485865 |
|  | 15494217 |
|  | 15498833 |
|  | 15522235 |
|  | 15522237 |
|  | 15528270 |
|  | 15532711 |
|  | 15546919 |
|  | 15583006 |
|  | 15588985 |
|  | 15618223 |
|  | 15623523 |
|  | 15623525 |
|  | 15637072 |
|  | 15647376 |
|  | 15688032 |
|  | 15689620 |
|  | 15695802 |
|  | 15696169 |
|  | 15735682 |
|  | 15746183 |
|  | 15774483 |
|  | 15777850 |
|  | 15782139 |
|  | 15788656 |
|  | 15818752 |
|  | 15835917 |
|  | 15845350 |
|  | 15853660 |
|  | 15872086 |
|  | 16002577 |
|  | 16027153 |
|  | 16055703 |
|  | 16158055 |
|  | 16158247 |
|  | 16163059 |
|  | 16168436 |
|  | 16186108 |
|  | 16260653 |
|  | 16317091 |
|  | 16322069 |
|  | 16371650 |
|  | 16407214 |
|  | 16412380 |
|  | 16414009 |
|  | 16440311 |
|  | 16441665 |
|  | 16465417 |
|  | 16492668 |
|  | 16523241 |
|  | 16569657 |
|  | 16619028 |
|  | 16636672 |
|  | 16690617 |
|  | 16837460 |
|  | 16844778 |
|  | 16862215 |
|  | 16891307 |
|  | 1689310 |
|  | 16894533 |
|  | 16959222 |
|  | 17018524 |
|  | 17056000 |
|  | 1713455 |
|  | 1717825 |
|  | 2173144 |
|  | 2457390 |
|  | 2506951 |
|  | 2581127 |
|  | 2582238 |
|  | 2981336 |
|  | 2996754 |
|  | 2996780 |
|  | 3299057 |
|  | 3917576 |
|  | 6092965 |
|  | 6304688 |
|  | 6333425 |
|  | 7510703 |
|  | 7513017 |
|  | 7513429 |
|  | 7516469 |
|  | 7517401 |
|  | 7518772 |
|  | 7519780 |
|  | 7521304 |
|  | 7527558 |
|  | 7529876 |
|  | 7530249 |
|  | 7532003 |
|  | 7534286 |
|  | 7537362 |
|  | 7537495 |
|  | 7539106 |
|  | 7542762 |
|  | 7678701 |
|  | 7682059 |
|  | 7691597 |
|  | 7997267 |
|  | 8054685 |
|  | 8070403 |
|  | 8253717 |
|  | 8272872 |
|  | 8510939 |
|  | 8621384 |
|  | 8626374 |
|  | 8632005 |
|  | 8635998 |
|  | 8647432 |
|  | 8647855 |
|  | 8657103 |
|  | 8657134 |
|  | 8681387 |
|  | 8702721 |
|  | 8761480 |
|  | 8766817 |
|  | 8797825 |
|  | 8805332 |
|  | 8816475 |
|  | 8845374 |
|  | 8849729 |
|  | 8876196 |
|  | 8878483 |
|  | 8885868 |
|  | 8900189 |
|  | 8910575 |
|  | 8918464 |
|  | 8940173 |
|  | 8941336 |
|  | 8953041 |
|  | 8978822 |
|  | 9012831 |
|  | 9016807 |
|  | 9020193 |
|  | 9024657 |
|  | 9096689 |
|  | 9118959 |
|  | 9174343 |
|  | 9178760 |
|  | 9182531 |
|  | 9195899 |
|  | 9208935 |
|  | 9261115 |
|  | 9278444 |
|  | 9388490 |
|  | 9507031 |
|  | 9560226 |
|  | 9566874 |
|  | 9584165 |
|  | 9621077 |
|  | 9632142 |
|  | 9655255 |
|  | 9687533 |
|  | 9701558 |
|  | 9710204 |
|  | 9748248 |
|  | 9763511 |
|  | 9786846 |
|  | 9792645 |
|  | 9819391 |
|  | 9819392 |
|  | 9837922 |
|  | 9837958 |
|  | 9867848 |
|  | 9880482 |
|  | 9924018 |
|  | 9988270 |
|  | 12824177 |
|  | 16446427 |
|  | 15815683 |
|  | 16109975 |
|  | 16603149 |
|  | 11780052 |
|  | 15489334 |
|  | 15851033 |
|  | 16479011 |
|  | 1691439 |
|  | 17038311 |
|  | 17046078 |
|  | 17062641 |
|  | 17135298 |
|  | 17192257 |
|  | 17215324 |
|  | 17224200 |
|  | 17307333 |
|  | 17317726 |
|  | 17325034 |
|  | 17443665 |
|  | 17510314 |
|  | 17537434 |
|  | 17537435 |
|  | 17574549 |
|  | 17620427 |
|  | 17641225 |
|  | 2681803 |
|  | 10549294 |
|  | 14600260 |
|  | 16835269 |
|  | 17010340 |
|  | 17114649 |
|  | 17118337 |
|  | 17322282 |
|  | 17360811 |
|  | 17449027 |
|  | 17558145 |
|  | 17569658 |
|  | 17569670 |
|  | 10082587 |
|  | 14529711 |
|  | 16157790 |
|  | 17021051 |
|  | 17136425 |
|  | 17156388 |
|  | 17252537 |
|  | 17335807 |
|  | 17401665 |
|  | 17446308 |
|  | 17629454 |
|  | 6253989 |
|  | 8402898 |
|  | 8805372 |

---

|  |  |
| --- | --- |
| Organism | Homo sapiens {Organism urn:agi-taxid:9606} |
|  | Mus musculus {Organism urn:agi-taxid:10090} |
|  | Rattus norvegicus {Organism urn:agi-taxid:10116} |
|  | Homo sapiens |
|  | Mus musculus |
|  | Rattus norvegicus |
|  | Bos taurus |
|  | Danio rerio |

---

|  |  |
| --- | --- |
| Mouse chromosome position | 2 78.35 cM |
|  | 2 89.0 cM |

---

|  |  |
| --- | --- |
| OMIM ID | 190090 |
|  | 114500 |
|  | 616937 |

---

|  |  |
| --- | --- |
| Rat chromosome position | 3q42 |

---

|  |  |
| --- | --- |
| Hugo ID | 11283 |
|  | HGNC:11283 |

---

|  |  |
| --- | --- |
| Human chromosome position | 20q11.23 |
|  | 20q12-q13 |

---

|  |  |
| --- | --- |
| Swiss-Prot Accession | P12931 |
|  | P12931.3 |
|  | F8WI90 |
|  | P05480 |
|  | P05480.4 |
|  | Q9WUD9 |
|  | Q9WUD9.4 |
|  | E1P5V4 |
|  | Q76P87 |
|  | Q86VB9 |
|  | Q9H5A8 |
|  | Q2M4I4 |
|  | G3V776 |
|  | Q45QJ2 |
|  | Q9JJ10 |
|  | Q80XU2 |
|  | Q71UK5 |
|  | Q9H7V3 |
|  | Q3UKD6 |
|  | SRC\_HUMAN |
|  | SRC\_MOUSE |
|  | Q2M4I4\_MOUSE |
|  | Q80XU2\_MOUSE |
|  | Q9JJ10\_RAT |
|  | SRC\_CHICK |
|  | NDEL1\_MOUSE |

---

|  |  |
| --- | --- |
| PIR ID | A26891 |
|  | A43610 |

---

|  |  |
| --- | --- |
| GenBank ID | NC\_000020 |
|  | NM\_005417 |
|  | NP\_005408 |
|  | XM\_017028026 |
|  | XP\_016883515 |
|  | XM\_017028024 |
|  | XP\_016883513 |
|  | XM\_017028025 |
|  | XP\_016883514 |
|  | XM\_011529013 |
|  | XP\_011527315 |
|  | NM\_198291 |
|  | NP\_938033 |
|  | XM\_017028027 |
|  | XP\_016883516 |
|  | NG\_023033 |
|  | AF077754 |
|  | AAC29427 |
|  | AH002989 |
|  | AAA60584 |
|  | AL034422 |
|  | AL133293 |
|  | AL390014 |
|  | AX405748 |
|  | CAD34855 |
|  | CH471077 |
|  | EAW76062 |
|  | EAW76063 |
|  | EAW76064 |
|  | EAW76065 |
|  | EAW76066 |
|  | EAW76067 |
|  | CS566144 |
|  | CAN37623 |
|  | KF456893 |
|  | M34469 |
|  | AAB59543 |
|  | X02647 |
|  | CAA26485 |
|  | X03995 |
|  | X03996 |
|  | X03997 |
|  | X03998 |
|  | X03999 |
|  | X04000 |
|  | AF272982 |
|  | AK024281 |
|  | BAB14871 |
|  | AK091756 |
|  | AW135290 |
|  | BC011566 |
|  | AAH11566 |
|  | BC051270 |
|  | AAH51270 |
|  | CD364525 |
|  | HY016645 |
|  | Z18364 |
|  | P12931 |
|  | NC\_000068 |
|  | XM\_011239404 |
|  | XP\_011237706 |
|  | XM\_006499066 |
|  | XP\_006499129 |
|  | XM\_011239402 |
|  | XP\_011237704 |
|  | XM\_011239403 |
|  | XP\_011237705 |
|  | XM\_006499063 |
|  | XP\_006499126 |
|  | NM\_009271 |
|  | NP\_033297 |
|  | NM\_001025395 |
|  | NP\_001020566 |
|  | XM\_006499065 |
|  | XP\_006499128 |
|  | AJ312258 |
|  | AJ312259 |
|  | AJ312260 |
|  | AJ312261 |
|  | AJ312262 |
|  | AJ312263 |
|  | AJ312264 |
|  | AJ313104 |
|  | AJ313176 |
|  | AJ313177 |
|  | AL672259 |
|  | AY902331 |
|  | AAX90616 |
|  | CH466551 |
|  | EDL06234 |
|  | M61221 |
|  | M61225 |
|  | AK017629 |
|  | AK146056 |
|  | BAE26865 |
|  | AK182969 |
|  | AK182983 |
|  | AK189521 |
|  | AK190301 |
|  | AK192339 |
|  | AK200868 |
|  | AK201096 |
|  | AK208623 |
|  | AK216314 |
|  | CD355175 |
|  | CX218263 |
|  | M17031 |
|  | AAA40135 |
|  | P05480 |
|  | NC\_005102 |
|  | XM\_017592067 |
|  | XP\_017447556 |
|  | XM\_017592066 |
|  | XP\_017447555 |
|  | XM\_017592070 |
|  | XP\_017447559 |
|  | XM\_017592065 |
|  | XP\_017447554 |
|  | XM\_017592069 |
|  | XP\_017447558 |
|  | XM\_017592068 |
|  | XP\_017447557 |
|  | XM\_017592071 |
|  | XP\_017447560 |
|  | XM\_008762388 |
|  | XP\_008760610 |
|  | XM\_008762387 |
|  | XP\_008760609 |
|  | XM\_017592072 |
|  | XP\_017447561 |
|  | NM\_031977 |
|  | NP\_114183 |
|  | AC\_000071 |
|  | AAHX01026092 |
|  | AC095852 |
|  | CH474005 |
|  | EDL96675 |
|  | EDL96676 |
|  | EDL96677 |
|  | EDL96678 |
|  | EDL96679 |
|  | EDL96680 |
|  | EDL96681 |
|  | AF130457 |
|  | AAD24180 |
|  | AF157016 |
|  | AAF80335 |
|  | DQ120509 |
|  | AAZ23848 |
|  | DQ120510 |
|  | AAZ23849 |
|  | Q9WUD9 |
|  | XM\_011529014 |
|  | XP\_011527316 |
|  | NC\_018931 |
|  | AMYH02037046 |
|  | K03212 |
|  | K03213 |
|  | K03214 |
|  | K03215 |
|  | K03216 |
|  | K03217 |
|  | K03218 |
|  | M16237 |
|  | M16243 |
|  | M16244 |
|  | M16245 |
|  | EU831426 |
|  | ACE87113 |
|  | EU831519 |
|  | ACE86438 |
|  | JF432365 |
|  | ADZ15582 |
|  | XM\_006499067 |
|  | XP\_006499130 |
|  | AC\_000024 |
|  | AAHY01012248 |
|  | AAHY01012249 |
|  | BC039953 |
|  | AAH39953 |
|  | XM\_008762389 |
|  | XP\_008760611 |
|  | AC\_000152 |
|  | ABBA01006617 |
|  | ABBA01006618 |
|  | ABBA01006619 |
|  | XM\_006499064 |
|  | XP\_006499127 |
|  | XM\_006235382 |
|  | XP\_006235444 |
|  | XM\_006235386 |
|  | XP\_006235448 |
|  | XM\_006235383 |
|  | XP\_006235445 |
|  | XM\_006235388 |
|  | XP\_006235450 |
|  | XM\_006235387 |
|  | XP\_006235449 |
|  | XM\_006235380 |
|  | XP\_006235442 |
|  | XM\_006235384 |
|  | XP\_006235446 |
|  | XM\_006235381 |
|  | XP\_006235443 |
|  | XM\_006235385 |
|  | XP\_006235447 |
|  | AABR06027223 |
|  | AABR06027224 |
|  | CAC10573 |
|  | CAC34523 |
|  | CAM16140 |
|  | CAM16141 |
|  | NT\_011362 |
|  | NW\_927339 |
|  | NW\_001838665 |
|  | AC\_000063 |
|  | Q71UK5 |
|  | Q9H7V3 |
|  | NT\_039207 |
|  | NW\_001030712 |
|  | Q2M4I4 |
|  | Q3UKD6 |
|  | Q80XU2 |
|  | NW\_001084817 |
|  | NW\_047659 |
|  | Q45QJ2 |
|  | Q9JJ10 |
|  | Q76P87 |
|  | BG868120 |
|  | CAI22921 |
|  | NW\_000179 |
|  | 123797574 |
|  | 4574719 |
|  | 71089987 |
|  | 71089989 |
|  | 8134704 |
|  | 81868594 |
|  | 8885998 |
|  | 123792899 |
|  | 123794673 |
|  | 125715 |
|  | 201057 |
|  | 25059027 |
|  | 62635494 |
|  | 74222098 |
|  | 81873417 |
|  | AW259666 |
|  | 10436623 |
|  | 10635153 |
|  | 119596470 |
|  | 119596472 |
|  | 119596473 |
|  | 125711 |
|  | 13374724 |
|  | 15079460 |
|  | 1848077 |
|  | 338460 |
|  | 3406615 |
|  | 55930980 |
|  | 74712826 |
|  | 74749849 |
|  | 74761511 |
|  | NT\_086910 |
|  | NC\_007311 |
|  | NW\_001493161 |
|  | XM\_615852 |
|  | XP\_615852 |
|  | 123912696 |
|  | 49609372 |
|  | 82086043 |
|  | 94574472 |
|  | AAI16545 |
|  | AJ620750 |
|  | BC116544 |
|  | BX548066 |
|  | CAF06181 |
|  | NC\_007134 |
|  | NM\_001003837 |
|  | NP\_001003837 |
|  | NW\_001512591 |
|  | Q1JPZ3 |
|  | Q6EWH0 |
|  | 119596468 |
|  | 119596469 |
|  | 119596471 |
|  | 123219085 |
|  | 123219086 |
|  | 148674287 |
|  | 149043101 |
|  | 149043102 |
|  | 149043103 |
|  | 149043104 |
|  | 149043105 |
|  | 149043106 |
|  | 149043107 |
|  | 99028937 |
|  | 126007504 |
|  | 34582248 |
|  | 99028938 |
|  | 325084 |
|  | Dr.2731 |
|  | ZDB-GENE-030131-3809 |
|  | dre:325084 |

---

|  |  |
| --- | --- |
| Swiss-Prot ID | SRC\_HUMAN |
|  | SRC\_MOUSE |
|  | SRC\_RAT |
|  | Q2M4I4\_MOUSE |
|  | Q9JJ10\_RAT |
|  | Q80XU2\_MOUSE |

---

|  |  |
| --- | --- |
| Cell Localization | Cell membrane |
|  | Mitochondrion inner membrane |
|  | Nucleus |
|  | Cytoplasm |
|  | Cytoskeleton |
|  | Perinuclear region |
|  | Cytoplasm, cytoskeleton |

---

|  |  |
| --- | --- |
| Ensembl ID | ENSG00000197122 |
|  | ENSP00000362680.2 |
|  | ENST00000373578.6 |
|  | ENSP00000362659.2 |
|  | ENST00000373558.2 |
|  | ENSMUSG00000027646 |
|  | ENSMUSP00000029175.7 |
|  | ENSMUST00000029175.13 |
|  | ENSMUSP00000090237.4 |
|  | ENSMUST00000092576.10 |
|  | ENSRNOG00000009495 |
|  | ENSRNOP00000071837.1 |
|  | ENSRNOT00000080516.1 |
|  | ENSRNOP00000012739.2 |
|  | ENSRNOT00000012739.4 |
|  | ENSP00000362680 |
|  | ENST00000373578 |
|  | ENSP00000350941 |
|  | ENST00000358208 |
|  | ENSMUSP00000029175 |
|  | ENSMUST00000029175 |
|  | ENSMUSP00000090237 |
|  | ENSMUST00000092576 |
|  | ENSRNOP00000071837 |
|  | ENSRNOT00000080516 |
|  | ENSRNOP00000012739 |
|  | ENSRNOT00000012739 |
|  | ENSP00000408503 |
|  | ENST00000445403 |

---

|  |  |
| --- | --- |
| MGI ID | MGI:98397 |
|  | 98397 |
|  | 2139334 |

---

|  |  |
| --- | --- |
| RGD ID | 620795 |

---

|  |  |
| --- | --- |
| TCDB ID | 8.A.23.1.12 |

---

|  |  |
| --- | --- |
| Unigene ID | Hs.195659 |
|  | Mm.22845 |
|  | Rn.112600 |
|  | Bt.61976 |
|  | Dr.2731 |

---

|  |  |
| --- | --- |
| Homologene ID | 21120 |

---

|  |  |
| --- | --- |
| Shape | Sickle-vertex |

---

|  |  |
| --- | --- |
| IPI ID | IPI00222801 |
|  | IPI00328867 |
|  | IPI00641230 |
|  | IPI00719950 |
|  | IPI00200886 |
|  | IPI00620622 |

---

|  |  |
| --- | --- |
| Microarray ID | 99388\_at |

---

|  |  |
| --- | --- |
| Homo sapiens Chromosome position | 20q12-q13 |

---

|  |  |
| --- | --- |
| Ensembl Transcript ID | ENST00000358208 |
|  | ENST00000373578 |
|  | ENST00000373567 |

---

|  |  |
| --- | --- |
| FunctionalClass | kinase |
|  | protein C-terminus binding |
|  | protein binding |
|  | protein kinase |
|  | protein serine/threonine kinase |
|  | protein-tyrosine kinase |
|  | ATP binding |
|  | nucleotide binding |
|  | transferase |
|  | SH2 domain binding |
|  | SH3/SH2 adaptor |

---

|  |  |
| --- | --- |
| Bos taurus Chromosome position | 13 |

---

|  |  |
| --- | --- |
| Mus musculus Chromosome position | 2 89.0 cM |
|  | 2 |

---

|  |  |
| --- | --- |
| KEGG pathway | Adherens junction |
|  | ErbB signaling pathway |
|  | Focal adhesion |
|  | Gap junction |
|  | GnRH signaling pathway |
|  | Tight junction |
|  | VEGF signaling pathway |

---

|  |  |
| --- | --- |
| Rattus norvegicus Chromosome position | 3q42 |

---

|  |  |
| --- | --- |
| Ensembl Gene ID | ENSG00000197122 |

---

|  |  |
| --- | --- |
| LocatorString | pmquery:egfr+transactivation |

---
